# The Effects of “Diet–Smoking–Gender” Three-Way Interactions on Cognitive Impairment among Chinese Older Adults

**DOI:** 10.3390/nu14102144

**Published:** 2022-05-20

**Authors:** Huashuai Chen, Xuxi Zhang, Qiushi Feng, Yi Zeng

**Affiliations:** 1 Business School, Xiangtan University, Xiangtan 411105, China; huashuai.chen@gmail.com; 2 Center for Healthy Aging and Development Studies, National School of Development, Peking University, Beijing 100871, China; zhangxuxi@pku.edu.cn; 3 Department of Sociology, Centre for Family and Population Research (CFPR), National University of Singapore, Singapore 119077, Singapore; socfq@nus.edu.sg; 4 Center for the Study of Aging and Human Development, Medical School of Duke University, Durham, NC 27710, USA

**Keywords:** cognitive impairment, diet, smoking, gender, three-way interaction, older adults, CLHLS

## Abstract

Investigations on gender variations in the risk factors of cognitive impairment are required to promote future precision medicine among older adults, as well as to contribute to a better understanding of the “male–female health-survival paradox”. With this study, we aimed to investigate the effects of “diet–smoking–gender” three-way interactions on cognitive impairments among Chinese older adults. We conducted a 16-year prospective cohort study among 15,953, 15,555, 16,849, 9716, 7116, and 13,165 older adults from the 2002, 2005, 2008–2009, 2011–2012, 2014, and 2017–2018 waves of the Chinese Longitudinal Healthy Longevity Survey (CLHLS), respectively. Cognitive impairment was measured by the Mini-Mental State Examination (MMSE). The dietary diversity score (DDS) was calculated using the CLHLS food frequency questionnaire. Generalized estimating equations (GEE) were used to assess the “diet–smoking–gender” three-way interaction effects on cognitive impairment across the six waves of CLHLS. We found that higher dietary diversity was associated with lower probability of cognitive impairment among older adults (OR = 0.92; 95%CI = 0.90, 0.98). However, smoking behavior may negatively influence the protective effect of higher dietary diversity on cognitive function among females (OR = 1.26; 95%CI = 1.07, 1.49). Our findings imply that we should take gender differences and lifestyle behaviors into consideration in implementing dietary interventions to improve cognitive function among older adults.

## 1. Introduction

Cognitive impairment is an age-related deterioration in cognitive functions with increasing difficulties with language and memory [1,2]. With the rapid aging of the population, cognitive impairment is anticipated to be a serious public health issue worldwide [2,3,4]. Diet and other lifestyle behaviors, such as smoking, have been proven to be associated with cognitive health among older adults in previous studies [5,6]. Due to the lack of an absolute medical cure for cognitive impairment [2,3], an increasing number of studies have focused on interventions in lifestyle behaviors, such as diet and smoking, to prevent and slow down the progression of cognitive impairment among older adults.

Dietary represents a possible preventative measure against cognitive impairment [5]. The cumulative effects of diverse diet components and dietary variety on cognitive function have recently drawn attention and might differ from the effect of a single nutrient or food item because of meals containing complex combinations of nutrients that tend to be linked and interact with one another [2,7,8,9]. Dietary diversity, as a rapid, user-friendly, and cost-effective tool, is widely used to evaluate overall diet quality [10,11,12]. This tool can provide an overall assessment of dietary behaviors, evaluate the effect of several food items simultaneously on health [10], bypass methodological problems of interactions, and facilitate a more comprehensive approach to disease control and prevention [11]. However, many studies on the effects of dietary diversity on cognitive function are limited due to small sample sizes, cross-sectional design, or short follow-up periods, and most current studies only focus on younger people in high-income countries [13,14].

Previous studies have also shown that smoking has adverse effects on health [15,16]. There are conflicting results regarding the association between smoking and cognitive impairment among older adults. Some studies found that smoking is associated with an increased risk of cognitive impairment [17,18], some found no association between smoking and risk of cognitive impairment [19,20], and, conversely, some found that smoking is associated with decreased risk of cognitive impairment [3,21]. These conflicting results might be caused by the complex interplay between smoking and other factors. Older adults with cognitive impairment may experience cholinergic deficits, which are characterized by reduced levels of acetylcholine and nicotinic receptors [22,23]. Nicotine might improve attention, learning, and memory by increasing acetylcholine release and elevating the number of nicotinic receptors [23,24]. These improvements may be opposed by the high oxidative stress caused by smoking [23]. Some studies also reported that smokers might have lower dietary intakes of certain antioxidants compared with nonsmokers [25,26]. However, the effect of the interplay between smoking and diet on cognitive impairment, as well as the mechanism of such an effect, is still unclear. The ongoing discussion on smoking–diet interactions is valuable for the development of practical lifestyle intervention protocols against cognitive impairment.

Moreover, the phenomenon of the “male–female health-survival paradox”, which states that females experience higher rates of health conditions but live longer than males, has been identified by many scholars [27,28]. Males and females differ in dementia prevalence [29,30,31] and also have distinctive lifestyles in terms of smoking and dietary habits. Studies on gender variations with regard to the risk factors of cognitive impairment can promote future precision health interventions among older adults and contribute to a better understanding of the “male–female health-survival paradox”. However, knowledge of gender differences with respect to the effects of diet, smoking, and diet–smoking interactions (i.e., “diet–smoking–gender” three-way interactions) on cognitive impairments is remarkably limited.

Therefore, in the current study, we aimed to investigate the effect of “diet–smoking–gender” three-way interactions on cognitive impairments among Chinese older adults aged 65 to 105 years old based on the 2002–2018 Chinese Longitudinal Healthy Longevity Survey (CLHLS), the largest dataset of oldest–old (with comparable young–old) cohorts in the world.

## 2. Materials and Methods

### 2.1. Data and Participants

The study data were derived from the Chinese Longitudinal Healthy Longevity Survey (CLHLS). Details of the survey design and participants have been described elsewhere [32,33]. Briefly, the CLHLS is a nationwide survey conducted in a randomly selected half of the counties and cities in 23 of the 31 provinces, covering about 85 percent of the total population of China [32]. In the current study, participants were enrolled and followed up in the CLHLS waves of 2002, 2005, 2008–2009, 2011–2012, 2014, and 2017–2018. We removed 3125 samples who did not answer the food frequency questions among the total 81,847 samples in the six waves. The average missing rate of the food frequency questionnaire is 3.8%, which is acceptable and was not expected to affect the estimation [34]. Finally, 15,953, 15,555, 16,849, 9716, 7116, and 13,165 effective samples from 2002, 2005, 2008–2009, 2011–2012, 2014, and 2017–2018 waves of CLHLS, respectively, were included in the analyses of the current study.

### 2.2. Measurements

#### 2.2.1. Cognitive Impairment

Cognitive impairment (yes vs. no) was assessed by the Chinese version of the Mini-Mental State Examination (MMSE) [35], which has been validated and applied widely by other scholars [32]; the Chinese version of MMSE was used across all CLHLS waves to ensure data comparability. The MMSE tests orientation, memory, attention, calculation, language, and written and visual construction, with possible scores ranging from 0 to 30, with a lower score representing poorer cognitive function [35]. We used education-specific cutoffs to define cognitive impairment based on the latest normative and validation study of the MMSE in the Chinese population [36]: less than or equal to 16 for those without schooling, less than or equal to 19 for those with 1–6 years of education, and less than or equal to 23 for those with more than 6 years of education.

#### 2.2.2. Dietary Diversity

According to previous literature [12,37,38], we adopted the food frequency questionnaire in the CLHLS to develop a dietary diversity score (DDS) to evaluate dietary diversity. Participants were asked to report their dietary frequencies of the major daily foods (meat, fish or seafood, eggs, beans, sugar, tea, and garlic), both at present and around age 60, which were recorded as “almost every day”, “not every day but at least once per week”, “not every week but at least once per month”, “not every month but occasionally”, or “rarely or never”. To avoid the possible effect of reverse causation on current DDS by cognitive condition, we only considered the past dietary frequencies around age 60 of the above seven foods. One DDS unit was defined as “almost every day” or “not every day but at least once per week” consumption of any of the above seven foods. DDS was thus considered a continuous variable, with a score range of 0–7, in which 7 represents the highest level of dietary diversity. Cronbach’s alpha coefficient of dietary frequencies of these seven foods in CLHLS was 0.7497, which is larger than 0.70 and means that DDS is reliable for measuring dietary frequency in our study. We also used threshold 4 to classify DDS as a dummy variable. Specifically, “low DDS” was defined as a DDS of 0–3, whereas “high DDS” was defined as a DDS of 4–7.

#### 2.2.3. Smoking Behavior

Smoking behavior was measured by the question of “Did you smoke in the past?”. If the answer to the question was “Yes”, then it was coded as 1; otherwise, it was coded as 0. Only smoking behavior in the past was considered because for two reasons. First, the DDS was measured according to past dietary frequencies around age 60. Second, there might be a reverse causation effect on current smoking behavior caused by cognitive condition.

#### 2.2.4. Covariates

We considered the following variables as covariates, all of which have been noted as major factors contributing to cognitive impairment in Chinese older adults [39,40]: age, years of schooling, family income per capita, residence status (urban vs. rural), region (eastern provinces vs. middle/western provinces), marital status (having spouse vs. no spouse), self-rated health status (good vs. poor/so so), number of coresident family members, and regular exercise at present (yes vs. no).

### 2.3. Statistical Analysis

The following model was used to explore the “diet–smoking–gender” 3-way interaction effects on cognitive impairment:

(1) $$\mathrm{logit}(p(Y_{i}))=\log\frac{p_{i}}{\left(1-p_{i}\right)}=\beta_{0}+\beta_{1}N_{i}+\beta_{2}B_{i}+\beta_{3}S_{i}+\beta_{4}N_{i}B_{i}+\beta_{5}N_{i}S_{i}+\beta_{6}B_{i}S_{i}+\beta_{7}N_{i}B_{i}S_{i}+\sum_{j}\alpha_{j}X_{ji}+u_{i}$$

where *Y_i_* is the status of cognitive impairment (yes vs. no); *P_i_* is the probability of cognitive impairment; *N_i_*, *B_i_*, and *S_i_* are the DDS, smoking frequency, and sex of the *i*th individual, respectively; *N_i_* × *B_i_*, *N_i_* × *S_i_*, and *B_i_* × *S_i_* are the three 2-way interaction items among *N_i_*, *B_i_*, and *S_i_*, respectively; *N_i_B_i_S_i_* is the 3-way interaction item; *X_ji_* is a vector of covariates of the *i*th individual; *P_i_*/(1 − *P_i_*) is the odds ratio (OR); and *u_i_* is the error term.

Model (1) shows whether a significant *N_i_B_i_S_i_* 3-way interaction effect on cognitive impairment exists or not. However, it is not possible to directly evaluate the effect of specific combinations of diet, smoking, and gender on cognitive impairment and its significance based on Model (1). Considering the difficulty to construct integrated binary variables based on continuous variable *N_i_* with dummies *B_i_* and *S_i_*, we transformed the continuous DDS into a dichotomous variable (low DDS vs. high DDS). Then, we used the following Model (2) to perform analyses of the integrated effects among diet, smoking, and gender on cognitive function of older adults:

(2) $$\mathrm{logit}(p(Y_{i}))=\log\frac{p_{i}}{\left(1-p_{i}\right)}=\gamma_{0}+\sum_{N_{i}=0}^{1}\sum_{B_{i}=0}^{1}\sum_{S_{i}=0}^{1}\gamma_{N_{i},B_{i},S_{i}}Z_{N_{i},B_{i},S_{i}}+\sum_{j}\alpha_{j}X_{ji}+u_{i}$$

where *Z_N,B,S_* is a series of dummy that which represent the combinations of the statuses of the dietary diversity score (*N* = 0, 1), ever smoker (*B* = 0, 1), and gender (*S* = 0, 1); and *X_ji_* is a vector of covariates of the *i*th individual. There are 8 (=2 × 2 × 2) combinations of the *N*, *B*, and gender statuses, and we used *Z_N=0,B=0,S=0_* (i.e., low DDS, non-smoker, and female) as the reference group. The coefficients *γ_N,B,S_* measure the odds ratio (OR) of the health outcome for the corresponding dummy variables, *Z_N,B,S_*.

Furthermore, we investigated the effect of each food involved in the DDS on cognitive function separately. We constructed 7 dichotomous variables of past dietary frequency, including meat, fish or seafood, eggs, beans, sugar, tea, and garlic. For each food, we classified answer categories of “not every week but at least once per month”, “not every month but occasionally”, and “rarely or never” into “low frequency” and answer categories of “almost every day” and “not every day but at least once per week” into “high frequency”. Taking “meat” as an example, we utilized Model (2) to perform an analyses of the integrated effects of meat, smoking, and gender on cognitive function of older adults, where *Z_N,B,S_* is a series of dummy variables that represent the combinations of the statuses of eating frequency of meat (*N* = 0, 1), ever smoker (*B* = 0, 1), and gender (*S* = 0, 1); and *X_ji_* is a vector of covariates of the *i*th individual. We used *Z_N=0,B=0,S=0_* (i.e., low frequency, non-smoker, and female) as the reference group.

Generalized estimating equations (GEE) were used in both Models (1) and (2) to assess the “diet–smoking–gender” 3-way interaction effects on cognitive impairment across the six waves of the CLHLS. As an expansion of generalized linear model (GLM), GEE can effectively model repeated categorical variables from longitudinal studies. We classified cognitive impairment as a dichotomous variable (1 = yes, 0 = no) as the dependent variable, so we used logit models and reported the odds ratios (ORs) and 95% confidence intervals (CIs) obtained from the model-estimated robust standard errors. Exchangeable correlation structure was used to account for subject-level repeated measures. We also tried the continuous variable of “cognitive function score (0–30)” as the dependent variable and obtained similar results (data not shown). All statistical analyses were performed using Stata version 14.0 (Stata/SE 14.0 for Windows, 4905 Lakeway Drive College Station, TX: Stata Corp LP).

## 3. Results

Table 1 presents the general characteristics of the sample at each wave of the CLHLS surveys in 2002, 2005, 2008, 2011, 2014, and 2018. The mean age of participants at each wave ranged from 85.31 to 86.93 years, and the proportion of female participants ranged from 53.9% to 57.3%. The proportion of participants with cognitive impairment at each wave ranged from 19.5% to 25.9%, and the proportion of participants with low DDS ranged from 29.1% to 69.7%. Table 2 presents the distribution of cognitive impairment and DDS by gender and smoking behavior.

Table 3 presents the results of “diet–smoking–gender” three-way interaction effects on cognitive impairment based on GEE logit regression models of six waves of the CLHLS (2002–2018), with DDS as a dichotomous variable. The results indicated that after adjusting for all the covariates:

(1) Among all samples, high DDS was negatively associated with cognitive impairment (OR = 0.94; 95%CI = 0.90, 0.98), and males had better cognitive function (OR = 0.81; 95%CI = 0.77, 0.86) compared with females.
(2) Among all samples, there was a significant “high DDS–smoking” interaction effect (OR = 1.11; 95%CI = 1.00, 1.24) on cognitive impairment. Regarding never-smoking participants, OR1 of cognitive impairment for high DDS versus low DDS was 0.92. Regarding participants who smoked in the past, OR2 of cognitive impairment for high DDS versus low DDS was 0.92 × 1.11 = 1.02 > 1.0 > OR1. Compared with never-smoking participants with low DDS, OR3 of cognitive impairment for participants who smoked in the past with high DDS was 0.92 × 0.92 × 1.11 = 0.94 > OR1. OR1, OR2, and OR3 indicate that “smoking in the past” may decrease the protective effect of high DDS on cognitive function.
(3) Among all samples, there was a significant “high DDS–smoking–gender” three-way interaction effect (OR = 0.80; 95%CI = 0.65, 1.00) on cognitive impairment.
(4) Among males, “high DDS–smoking” two-way interaction effects on cognitive function were not significant, which indicates that high DDS was always a protective factor with respect to cognitive function among males, regardless of their smoking behavior in the past.
(5) Among females, there was a significant “high DDS–smoking” interaction effect (OR = 1.26; 95%CI = 1.07, 1.49) on cognitive impairment. Regarding never-smoking females, OR4 of cognitive impairment for high DDS versus low DDS was 0.92. Regarding females who smoked in the past, OR5 of cognitive impairment for high DDS versus low DDS was 0.92 × 1.26 = 1.16 > 1.0 > OR4. Compared with never-smoking females with low DDS, OR6 of cognitive impairment for females who smoked in the past with high DDS was 0.92 × 0.87 × 1.26 = 1.01 > 1.0 > OR4. OR4, OR5, and OR6 indicate that “smoking in the past” may offset the protective effect of high DDS on cognitive function among females.

**Figure 1:**
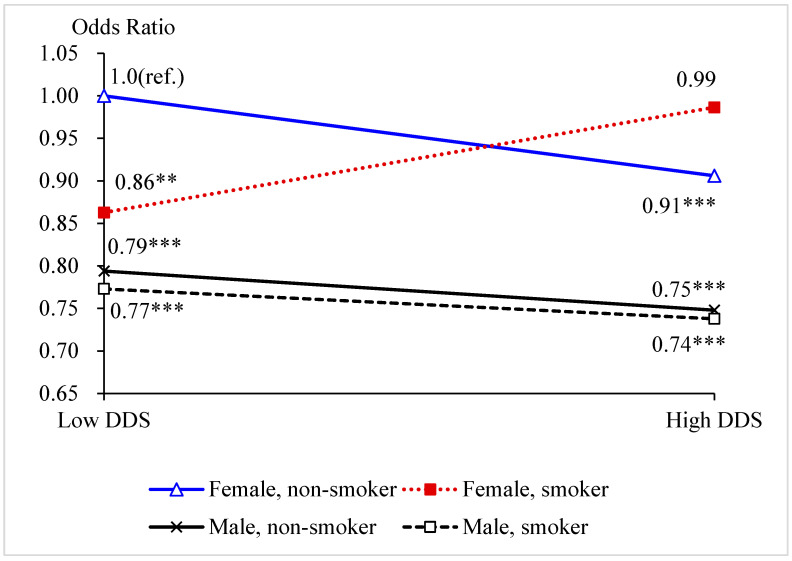
Odds ratios of the combinations of the statuses of DDS, smoking, and gender on cognitive impairment. Note: ** *p* < 0.05. *** *p* < 0.01.

Supplementary Table S1 presents the results of the integrated model of “DDS–smoking–gender” three-way interaction effects on cognitive impairment. We modified the “DDS–smoking–gender” three-way interaction model from Model (1) to obtain integrated Model (2), in which we defined eight dummy variables that represent the combinations of the statuses of the dietary diversity score (*N* = 0, 1), ever smoker (*B* = 0, 1), and gender (*S* = 0, 1) and used the combination “low DDS, non-smoker, female” as the reference group. Figure 1 presents the curves of the ORs derived from Supplementary Table S1.

Among males, the two curves were relatively parallel, with a decreasing trend, which suggests that there was no interaction between DDS and smoking behavior (see Figure 1). “Smoking in the past” does not influencing the protective effect of high DDS on cognitive function. Among both never smoker and ever smoker, males with high DDS had a lower risk of experiencing cognitive impairment than those with low DDS. Among females, the crossed curves indicate there an interaction between DDS and smoking behavior. Among never smoker, females with high DDS had a lower risk of experiencing cognitive impairment than those with low DDS. However, among ever smokers, females with high DDS had a higher risk than those with low DDS. These results are consistent with the results we found based on Table 3, which imply that “smoking in the past” may offset the protective effect of high DDS on cognitive function among females only.

Furthermore, we also observed that among females with high DDS, non-smokers had lower risk of experiencing cognitive impairment than ever smokers. However, among females with low DDS, non-smokers had a significantly higher risk of experiencing cognitive impairment than ever smokers.

Supplementary Table S2 presents the results of “dietary–smoking–gender” three-way interaction effects on cognitive impairment of older adults from the GEE logit regression models of six waves of the CLHLS (2002–2018), with eating frequency of each of seven foods (meat, fish or seafood, eggs, beans, sugar, tea, and garlic) as the diet dichotomous variable. The results indicate that after adjusting for all the covariates:

(1) Among all samples, we found that a high dietary frequency of “meat” (OR = 0.95; 95%CI = 0.90, 0.99), “fish or seafood” (OR = 0.95; 95%CI = 0.91, 1.00), “beans” (OR = 0.96; 95%CI = 0.91, 1.00), “tea” (OR = 0.92; 95%CI = 0.87, 0.96), and “garlic” (OR = 0.96; 95%CI = 0.92, 1.00) were associated with a lower risk of cognitive impairment.
(2) Among all samples, there was a significant “high dietary frequency–smoking–gender” three-way interaction effect on cognitive impairment with “meat” or “fish or seafood” as the diet dichotomous variable.
(3) Among males, the “high dietary frequency–smoking” interaction effect on cognitive impairment with “meat” as the diet dichotomous variable was not significant, which indicates that high dietary frequency of “meat” was always a protective factor with respect to cognitive function among males, regardless of their smoking behavior in the past. Regarding “fish or seafood”, there was a significant “high dietary frequency–smoking” interaction effect (OR = 0.89; 95%CI = 0.77, 1.02) on cognitive impairment. For never-smoking males, the OR of cognitive impairment for high dietary frequency versus low dietary frequency was 0.99. For males who smoked in the past, the OR of cognitive impairment for high dietary frequency versus low dietary frequency was 0.99 × 0.89 = 0.88. Compared with never-smoking males with low dietary frequency, the OR of cognitive impairment for males who smoked in the past with high dietary frequency was 0.99 × 1.02 × 0.89 = 0.90. These results indicate that high dietary frequency of “fish or seafood” was a protective factor with respect to cognitive function among both never-smoking males and males who smoked in the past.
(4) Among females, regarding “meat”, there was a significant “high dietary frequency–smoking” interaction effect (OR = 1.17; 95%CI = 0.99, 1.38) on cognitive impairment. For never-smoking females, the OR of cognitive impairment for high dietary frequency versus low dietary frequency was 0.93. For females who smoked in the past, the OR of cognitive impairment for high dietary frequency versus low dietary frequency was 0.93 × 1.17 = 1.09 > 0.93. Compared with never-smoking females with low dietary frequency, the OR of cognitive impairment for females who smoked in the past with high dietary frequency was 0.93 × 0.90 × 1.17 > 0.93. These estimates indicate that “smoking in the past” may offset the protective effect of high dietary frequency of “meat” on cognitive function among females. Similarly, the results also indicate that “smoking in the past” may offset the protective effect of high dietary frequency of “fish or seafood” on cognitive function among females.

## 4. Discussion

In the current study, we examined the effect of “diet–smoking–gender” three-way interactions on cognitive impairments based on analyses of the large datasets of a longitudinal study among Chinese older adults. We found that higher dietary diversity was associated with a lower probability of cognitive impairment among older adults. However, smoking in the past may negatively influence the protective effect of higher dietary diversity on cognitive function. Among females, smoking in the past offset the protective effect of higher dietary diversity on cognitive function. However, dietary diversity was always a protective factor with respect to cognitive function among males.

Previous studies reported that there was a positive association between higher dietary diversity and cognitive function [2,13,41,42], which is consistent with our findings. We further explored the effect of each individual food separately, and we found that high dietary frequency of “meat”, “fish or seafood”, “beans”, “tea”, and “garlic” were associated with a lower risk of cognitive impairment. A cross-sectional study among US adults aged 60 years and older also found that protein intake from meat was positively associated with cognitive function [43]. Several epidemiological studies among older adults found significant associations between seafood and a lower risk of cognitive decline [44,45]. A systematic review of 21 studies reported that green tea might have positive effects on reducing anxiety and improving memory, attention, and brain function [46]. We hypothesized that the underlying mechanism of the positive effect on cognitive function is that the nutrition intake may improve cognitive function by modifying the metabolism pathways. For example, raw garlic contains many distinct chemical components, including allicin [47]. Experimental evidence suggests that allicin can improve cognitive function by strengthening antioxidant pathways [48]. Additionally, food such as beans is rich in lecithin, which is a precursor to the neurotransmitter acetylcholine and has been shown to improve brain and memory performance [49]. Food such as fish or other seafood is rich in docosahexaenoic acid (DHA), a bioactive compound that has been proven to be beneficial to heart, cardiovascular, and brain function [45]. However, the mechanism of cumulative effects of diverse diet components and dietary variety on cognitive function is still unclear, and more experimental studies are needed to confirm our hypotheses.

Furthermore, we also found interactions between smoking in the past and higher dietary diversity on cognitive impairment; smoking behavior may negatively influence the effect of higher dietary diversity on cognitive function. We hypothesized the underlying mechanism of the interactions between smoking behavior and higher dietary diversity on cognitive function is that nicotine intake in smokers might have an adverse effect on the release of some neurotransmitters [50], and such a negative influence may neutralize or even reverse the positive effect of dietary diversity on cognitive function. Additionally, we found gender variations in interactions between smoking in the past and dietary diversity with respect to cognitive impairment. Our results indicate that smoking in the past may influence the effect of higher dietary diversity on cognitive function among females only. Previous studies also reported differences in cognitive function between males and females [13,51]. Genetic variations between females and males, as well as gender variations in the pattern of lifestyle behaviors, are potential causes of such differences [52]. We further investigated the “dietary–smoking–gender” three-way interaction effects of each food separately, and we found that “meat” and “fish or seafood” contributed most to the gender variations in interactions between smoking in the past and dietary diversity with respect to cognitive impairment. However, the underlying causes or mechanisms of gender differences are unclear, and more studies are needed. We recommend further investigation of potential gender differences in interactions between smoking behavior and dietary diversity with respect to cognitive function in order to confirm our findings and explore the underlying mechanism.

Finally, we observed that among females with high dietary diversity, those who had smoked in the past had a higher risk of experiencing cognitive impairment than never smokers. In contrast, among females with low dietary diversity, those who had smoked in the past had a lower risk of experiencing cognitive impairment than never smokers. Previous studies have also shown conflicting results with respect to the association between smoking in the past and cognitive function among older adults [3,53]. Some studies found that those who had smoked in the past had better cognitive function than never smokers among older adults [53,54]. Some reported no association between smoking in the past and cognitive function [3]. Conversely, some found that those who had smoked in the past had a higher risk of cognitive decline [18,55]. However, the potential mechanism is still unclear, and studies focusing on gender variations in the association between smoking behavior in the past and cognitive function are relatively limited. In our study, the sample size of females who had smoked in the past was much smaller than other groups, and we cannot conclude whether this relationship is caused or affected by other unmeasured confounding factors. Therefore, we recommend more future studies to confirm this finding and explore the potential mechanism.

To the best of our knowledge, this is the first study exploring the effect of “diet–smoking–gender” three-way interactions on cognitive impairment among older adults. Strengths of this study also include the large study size with a nationally representative sample of Chinese older adults and the longitudinal design of the six waves from 2002 to 2018. However, there are still some limitations of the current study. First, we measured the dietary diversity only due to the availability of questions in CLHLS. We recommend further studies to investigate the frequency and amount of nutrient intake to provide more comprehensive dietary suggestions. Second, we could not examine the hypothesized underlying mechanism of “diet–smoking–gender” three-way interactions with respect to cognitive impairments, and we recommend future experimental studies to investigate this mechanism. Third, there might be bidirectional associations between dietary diversity or smoking and cognitive function, and the cognitive function of older adults might influence their dietary diversity or smoking behavior. However, we only adopted the past DDS at around 60 years and smoking in the past in the current study in order to reduce the possible effect of reverse causation on current DDS or current smoking by cognitive condition.

## 5. Conclusions

We found a “diet–smoking–gender” three-way interaction with respect to cognitive impairment among older adults, which shows that smoking behavior may offset the protective effect of higher dietary diversity on cognitive function but may not influence the effect of higher dietary diversity on cognitive function among males. Our findings imply that we should take gender differences and lifestyle behaviors into consideration before implementing dietary interventions to promote cognitive function among older adults. We recommend policymakers pay more attention to gender differences among older adults and consider strategies to support the development of personalized precision diet interventions. However, due to the complexity of diet and the unclear mechanism of cumulative effects of diverse diet components on cognitive function, as well as their interactions with gender and smoking, our results are far from conclusive, and more studies are needed to provide scientific evidence for the future development of precision diet interventions. We recommend further investigation on potential gender differences in interactions between smoking behavior and diet with respect to cognitive function, as well as the underlying mechanisms.

## Figures and Tables

**Table 1:** Characteristics of the study samples in each of the six waves of CLHLS 2002–2018.

Characteristic	2002	2005	2008–2009	2011–2012	2014	2017–2018
	*n* = 15,953	*n* = 15,555	*n* = 16,849	*n* = 9716	*n* = 7116	*n* = 13,165
Cognitive Impairment (%)						
Yes	4133 (25.9)	2869 (20.0)	3559 (23.2)	1974 (21.4)	1312 (19.5)	2680 (21.3)
No	11,804 (74.1)	11,484 (80.0)	11,762 (76.8)	7254 (78.6)	5423 (80.5)	9897 (78.7)
DDS						
DDS (0–7), mean (SD)	4.39 (1.99)	4.47 (1.96)	2.42 (1.97)	2.49 (1.97)	2.58 (2.00)	2.84 (1.98)
High DDS (%)	11,083 (69.5)	11,034 (70.9)	5097 (30.3)	3086 (31.8)	2375 (33.4)	5259 (39.9)
Low DDS (%)	4870 (30.5)	4521 (29.1)	11,752 (69.7)	6630 (68.2)	4741 (66.6)	7906 (60.1)
Ever smoker (%)						
Yes	5373 (33.7)	5231 (33.6)	5271 (31.3)	3048 (31.4)	2091 (29.4)	3719 (28.2)
No	10,580 (66.3)	10,324 (66.4)	11,578 (68.7)	6668 (68.6)	5025 (70.6)	9446 (71.8)
Gender (%)						
Male	6807 (42.7)	6665 (42.8)	7187 (42.7)	4378 (45.1)	3283 (46.1)	5632 (42.8)
Female	9146 (57.3)	8890 (57.2)	9662 (57.3)	5338 (54.9)	3833 (53.9)	7533 (57.2)
Covariates						
Age, mean (SD)	86.28 (11.7)	86.13 (11.7)	86.93 (11.8)	85.75 (11.4)	85.31 (10.7)	85.46 (11.9)
Region (%)						
East province	7663 (48.0)	7040 (45.3)	7796 (46.3)	4645 (47.8)	3452 (48.5)	6754 (51.3)
Middle/west	8290 (52.0)	8515 (54.7)	9053 (53.7)	5071 (52.2)	3664 (51.5)	6411 (48.7)
Residence (%)						
Urban area	7339 (46.0)	6929 (44.5)	6628 (39.3)	4601 (47.4)	3193 (44.9)	7568 (57.5)
Rural area	8614 (54.0)	8626 (55.5)	10,221 (60.7)	5115 (52.6)	3923 (55.1)	5597 (42.5)
Married/partnered (%)						
Yes	5017 (31.4)	5070 (32.6)	5481 (32.5)	3690 (38.0)	2778 (39.0)	5363 (40.7)
No	10,936 (68.6)	10,485 (67.4)	11,368 (67.5)	6026 (62.0)	4338 (61.0)	7802 (59.3)
Years of schooling, mean (SD)	2.02 (3.50)	2.11 (3.53)	2.04 (3.42)	2.29 (3.51)	2.38 (3.47)	3.16 (4.23)
# of family members, mean (SD)	2.95 (1.87)	2.82 (1.73)	2.68 (1.68)	2.61 (1.75)	2.37 (1.61)	2.37 (1.61)
Log of income per capita, mean (SD)	7.59 (1.23)	7.86 (1.45)	8.32 (1.31)	8.64 (1.61)	8.95 (1.48)	9.22 (1.82)
Poor self-rated health (%)						
Yes	2466 (15.5)	2351 (15.1)	2407 (14.3)	1556 (16.0)	1001 (14.1)	1732 (13.2)
No	13,487 (84.5)	13,204 (84.9)	14,442 (85.7)	8160 (84.0)	6115 (85.9)	11,433 (86.8)
Regular exercise (%)						
Yes	5919 (37.1)	5434 (34.9)	5042 (29.9)	2521 (25.9)	1910 (26.8)	4189 (31.8)
No	10,034 (62.9)	10,121 (65.1)	11,807 (70.1)	7195 (74.1)	5206 (73.2)	8976 (68.2)

Note: DDS = dietary diversity score (0–7); “low DDS” is defined as a DDS in the range of 1–3, whereas “high DDS” is defined as a DDS in the range of 4–7.

**Table 2:** Distribution of cognitive impairment and dietary diversity score by gender and smoking behavior, combined by the sex waves of the 2002–2018 CLHLS.

Items	Male		Female	
	Non-Smoker	Smoker	Non-Smoker	Smoker
	*n* = 13,617	*n* = 19,175	*n* = 36,709	*n* = 4650
Cognitive impairment				
Yes (%)	2465 (18.1)	2685 (14.0)	10,107 (27.5)	1270 (27.3)
No (%)	11,152 (81.9)	16,490 (86.0)	26,602 (72.5)	3380 (72.7)
Dietary diversity score (DDS)				
DDS (0–7), mean (SD)	3.50 (2.17)	3.74 (2.14)	3.10 (2.16)	3.37 (2.17)
High DDS (%)	7079 (52.0)	10,759 (56.1)	16,279 (44.3)	2301 (49.5)
Low DDS (%)	6538 (48.0)	8416 (43.9)	20,430 (55.7)	2349 (50.5)

**Table 3:** “DDS–smoking–gender” three-way interaction effects on cognitive impairment of older adults from GEE logit regression models of six waves of the CLHLS (2002–2018) with DDS as a dichotomous variable.

	(1) All Samples	(2) All Samples	(3) All Samples	(4) Males Only	(5) Females Only
	OR [95%CI]	OR [95%CI]	OR [95%CI]	OR [95%CI]	OR [95%CI]
High DDS (low DDS *)	0.94 [0.90, 0.98] ***	0.92 [0.87, 0.98] ***	0.91 [0.85, 0.96] ***	0.91 [0.82, 1.02] *	0.92 [0.87, 0.98] **
Ever smoking (no *)	0.97 [0.92, 1.03]	0.92 [0.83, 1.01] *	0.86 [0.77, 0.97] **	0.96 [0.87, 1.06]	0.87 [0.77, 0.97] **
Male (female *)	0.81 [0.77, 0.86] ***	0.82 [0.76, 0.88] ***	0.79 [0.73, 0.86] ***	--	--
Interaction items					
High DDS × smoking		1.11 [1.00, 1.24] *	1.26 [1.07, 1.49] ***	1.01 [0.88, 1.16]	1.26 [1.07, 1.49] ***
High DDS × male		0.98 [0.88, 1.08]	1.04 [0.92, 1.17]	--	--
Smoking× male		1.02 [0.91, 1.14]	1.13 [0.97, 1.31]	--	--
High DDS × smoking × male			0.80 [0.65, 1.00] **	--	--
Covariates					
Age	1.12 [1.12, 1.12] ***	1.12 [1.12, 1.12] ***	1.12 [1.12, 1.12] ***	1.11 [1.11, 1.12] ***	1.12 [1.12, 1.13] ***
East China (middle/west *)	0.95 [0.91, 1.00] **	0.95 [0.91, 1.00] **	0.95 [0.91, 1.00] **	0.98 [0.91, 1.05]	0.94 [0.89, 0.99] **
Urban residence (rural *)	0.98 [0.93, 1.02]	0.98 [0.93, 1.02]	0.98 [0.93, 1.02]	1.01 [0.94, 1.09]	0.97 [0.91, 1.02]
Married (others *)	0.78 [0.73, 0.83] ***	0.78 [0.73, 0.83] ***	0.78 [0.73, 0.83] ***	0.79 [0.73, 0.86] ***	0.73 [0.66, 0.81] ***
Years of schooling	1.02 [1.01, 1.03] ***	1.02 [1.01, 1.03] ***	1.02 [1.01, 1.03] ***	1.02 [1.01, 1.03] ***	1.01 [1.00, 1.03] **
Number of family members	1.03 [1.01, 1.04] ***	1.03 [1.01, 1.04] ***	1.03 [1.01, 1.04] ***	1.02 [1.00, 1.04] **	1.03 [1.01, 1.04] ***
Log of income per capita	0.91 [0.90, 0.93] ***	0.91 [0.90, 0.93] ***	0.91 [0.90, 0.93] ***	0.91 [0.89, 0.93] ***	0.92 [0.90, 0.93] ***
Poor self-rated health (good ^#^)	1.97 [1.87, 2.08] ***	1.97 [1.87, 2.08] ***	1.97 [1.87, 2.08] ***	2.22 [2.03, 2.43] ***	1.84 [1.72, 1.97] ***
Regular exercise (no ^#^)	0.76 [0.73, 0.80] ***	0.76 [0.73, 0.80] ***	0.76 [0.73, 0.80] ***	0.75 [0.70, 0.81] ***	0.77 [0.73, 0.83] ***
Waves (2002 ^#^)					
2005	0.80 [0.75, 0.85] ***	0.80 [0.75, 0.85] ***	0.80 [0.75, 0.85] ***	0.76 [0.68, 0.84] ***	0.83 [0.77, 0.89] ***
2008	0.91 [0.86, 0.98] ***	0.91 [0.86, 0.97] ***	0.91 [0.86, 0.97] ***	0.83 [0.75, 0.93] ***	0.96 [0.89, 1.04]
2011	0.96 [0.89, 1.03]	0.96 [0.89, 1.03]	0.96 [0.89, 1.03]	0.82 [0.72, 0.93] ***	1.05 [0.95, 1.15]
2014	0.96 [0.88, 1.05]	0.96 [0.88, 1.05]	0.96 [0.88, 1.05]	0.91 [0.79, 1.04]	1.00 [0.89, 1.11]
2018	1.00 [0.93, 1.08]	1.00 [0.93, 1.07]	1.00 [0.93, 1.07]	0.96 [0.85, 1.08]	1.03 [0.94, 1.12]

Note: GEE = generalized estimation equation; high DDS = DDS in the range of 4–7; low DDS = DDS in the range of 0–3; OR = odds ratio; CI = confidence interval; ^#^ denotes the reference group. Dependent variable “cognitive impairment (no^#^)” uses cutoffs 16/17, 19/20, and 23/24 for those without schooling, 1–6 years of education, and more than 6 years of education, respectively. Odds ratios of covariates and waves are not listed. * *p* < 0.10. ** *p* < 0.05. *** *p* < 0.01.

## Data Availability

Publicly available datasets were analyzed in this study. This data can be found here: https://opendata.pku.edu.cn/dataverse/CHADS (accessed on 6 March 2022).

## Supplementary Materials

The following supporting information can be downloaded at: https://www.mdpi.com/article/10.3390/nu14102144/s1, Table S1: Integrated model of “DDS × smoking × gender” three-way interaction effects on cognitive impairment. Table S2: “Dietary-Smoking-Gender” 3-way interaction effects on cognitive impairment of older adults from the GEE Logit regression models in six waves of CLHLS (2002-2018) with eating frequency of each of the 7 foods (meat, fish and seafood, eggs, beans, sugar, tea, and garlic) as a dichotomous variable of dietary respectively.
