# Supplementary material for: The Effects of “Diet–Smoking–Gender” Three-Way Interactions on Cognitive Impairment among Chinese Older Adults
Source: Nutrients. 2022 May 20;14(10):2144. doi: 10.3390/nu14102144 (PMC9147822; doi:10.3390/nu14102144)
Supplement: Supplementary file 1 [file nutrients-14-02144-s001.zip › nutrients-1648121-supplementary.pdf]

**Supplementary Table S1** Integrated model of "DDS x Smoking x Gender" 3-way interaction effects on cognitive impairment

| Combination of DDS*Smoking*Gender | OR[95%CI] |
|---|---|
| Female, Non-smoker, Low DDS | 1.00(ref.) |
| Female, Non-smoker, high DDS | 0.91[0.85,0.96]*** |
| Female, Smoker, Low DDS | 0.86[0.77,0.97]** |
| Female, Smoker, high DDS | 0.99[0.87,1.11] |
| Male, Non-smoker, Low DDS | 0.79[0.73,0.86]*** |
| Male, Non-smoker, high DDS | 0.75[0.68,0.82]*** |
| Male, Smoker, Low DDS | 0.77[0.71,0.84]*** |
| Male, Smoker, high DDS | 0.74[0.68,0.80]*** |

Note: GEE = Generalized estimation equation; DDS = Dietary Diversity Score (0-7); High DDS = DDS ranged 4-7; Low DDS = DDS ranged 0-3; OR = Odds Ratio; CI = confidence interval; Covariates are the same as in Table 2. The ORs of covariates and waves are not listed. *p < .10. **p < .05. ***p < .01.

**Supplementary Table S2** "Dietary-Smoking-Gender" 3-way interaction effects on cognitive impairment of older adults from the GEE Logit regression models in six waves of CLHLS (2002-2018) with eating frequency of each of the 7 foods (meat, fish and seafood, eggs, beans, sugar, tea, and garlic) as a dichotomous variable of dietary respectively.

| Past dietary frequency around age 60 | (1) Meat | (2) Fish or seafood | (3) Eggs | (4) Beans | (5) Sugar | (6) Tea | (7) Garlic |
|---|---|---|---|---|---|---|---|
| | OR[95%CI] | OR[95%CI] | OR[95%CI] | OR[95%CI] | OR[95%CI] | OR[95%CI] | OR[95%CI] |
| **(1) All samples** | | | | | | | |
| High frequency (Low#) | 0.95[0.90,0.99]** | 0.95[0.91,1.00]** | 0.98[0.94,1.02] | 0.96[0.91,1.00]* | 1.06[1.01,1.11]** | 0.92[0.87,0.96]*** | 0.96[0.92,1.00]* |
| Ever smoking (no#) | 0.97[0.92,1.03] | 0.97[0.92,1.03] | 0.97[0.92,1.02] | 0.97[0.92,1.02] | 0.97[0.92,1.03] | 0.98[0.93,1.03] | 0.97[0.92,1.03] |
| Male (Female#) | 0.81[0.77,0.86]*** | 0.81[0.77,0.86]*** | 0.81[0.77,0.86]*** | 0.81[0.77,0.86]*** | 0.81[0.77,0.86]*** | 0.82[0.77,0.86]*** | 0.81[0.77,0.86]*** |
| Covariates | √ | √ | √ | √ | √ | √ | √ |
| Waves (2002#) | √ | √ | √ | √ | √ | √ | √ |
| **(2) All samples** | | | | | | | |
| High frequency (Low#) | 0.91[0.86,0.97]*** | 0.92[0.86,0.97]*** | 0.95[0.89,1.00]* | 0.93[0.88,0.99]** | 1.03[0.97,1.10] | 0.95[0.89,1.01]* | 0.97[0.92,1.03] |
| Ever smoking (no#) | 0.89[0.80,1.00]* | 0.86[0.77,0.96]*** | 0.89[0.79,1.01]* | 0.89[0.78,1.01]* | 0.92[0.83,1.03] | 0.96[0.87,1.07] | 0.97[0.86,1.09] |
| Male (Female#) | 0.77[0.71,0.84]*** | 0.77[0.71,0.84]*** | 0.78[0.71,0.85]*** | 0.79[0.73,0.87]*** | 0.78[0.72,0.84]*** | 0.83[0.77,0.90]*** | 0.81[0.74,0.88]*** |
| High frequency×Smoking | 1.17[1.00,1.38]* | 1.33[1.13,1.58]*** | 1.15[0.97,1.35] | 1.15[0.98,1.36]* | 1.11[0.94,1.31] | 0.98[0.82,1.17] | 0.97[0.83,1.15] |
| High frequency×Male | 1.09[0.97,1.23] | 1.11[0.99,1.25]* | 1.07[0.95,1.20] | 1.03[0.92,1.16] | 1.10[0.97,1.24] | 0.91[0.81,1.04] | 0.99[0.88,1.11] |
| Smoking×Male | 1.12[0.96,1.30] | 1.20[1.04,1.39]** | 1.09[0.93,1.28] | 1.09[0.92,1.28] | 1.09[0.95,1.26] | 1.02[0.89,1.18] | 1.03[0.88,1.20] |
| High frequency×Smoking×Male | 0.82[0.66,1.02]* | 0.67[0.54,0.83]*** | 0.88[0.71,1.10] | 0.89[0.72,1.11] | 0.84[0.68,1.05] | 1.04[0.83,1.31] | 0.99[0.80,1.23] |
| Covariates | √ | √ | √ | √ | √ | √ | √ |
| Waves (2002#) | √ | √ | √ | √ | √ | √ | √ |
| **(3) Males only** | | | | | | | |
| High frequency (Low#) | 0.97[0.87,1.08] | 0.99[0.89,1.10] | 0.99[0.89,1.10] | 0.94[0.84,1.04] | 1.11[0.99,1.23]* | 0.85[0.76,0.95]*** | 0.95[0.85,1.05] |
| Ever smoking (no#) | 0.99[0.89,1.09] | 1.02[0.93,1.11] | 0.96[0.86,1.07] | 0.95[0.85,1.06] | 0.99[0.90,1.08] | 0.97[0.88,1.07] | 0.99[0.89,1.09] |
| High frequency*Smoking | 0.96[0.83,1.10] | 0.89[0.77,1.02]* | 1.01[0.88,1.16] | 1.02[0.89,1.18] | 0.93[0.81,1.08] | 1.03[0.89,1.18] | 0.96[0.84,1.10] |
| Covariates | √ | √ | √ | √ | √ | √ | √ |

| | | | | | | | |
|---|---|---|---|---|---|---|---|
| Waves (2002#) | √ | √ | √ | √ | √ | √ | √ |
| **(4) Females only** | | | | | | | |
| High frequency (Low#) | 0.93[0.87,0.98]** | 0.93[0.88,0.99]** | 0.96[0.90,1.02] | 0.95[0.89,1.01]* | 1.05[0.98,1.11] | 0.96[0.90,1.02] | 0.98[0.92,1.04] |
| Ever smoking (no#) | 0.90[0.80,1.00]* | 0.86[0.77,0.96]*** | 0.89[0.79,1.01]* | 0.89[0.78,1.01]* | 0.92[0.83,1.03] | 0.97[0.87,1.07] | 0.97[0.86,1.09] |
| High frequency×Smoking | 1.17[0.99,1.38]* | 1.33[1.13,1.58]*** | 1.15[0.97,1.35] | 1.15[0.97,1.36]* | 1.11[0.94,1.31] | 0.98[0.82,1.17] | 0.97[0.83,1.15] |
| Covariates | √ | √ | √ | √ | √ | √ | √ |
| Waves (2002#) | √ | √ | √ | √ | √ | √ | √ |

*Note*: GEE = Generalized estimation equation; OR = Odds Ratio; CI = confidence interval; #Denotes the reference group. Dependent variable "Cognitive Impairment (no#)" uses cutoffs 16/17, 19/20, 23/24 for those without schooling, 1-6 years of education, and more than 6 years education, respectively. Covariates are the same as that of Table 3 including Age, East China (Middle/West#), Urban residence (Rural#), Married (Others#), Years of schooling, Number of family members, Log of income per capita, Poor self-rated health(good#) and Regular exercise (no#). Odds ratios of covariates and waves are not listed. *p < .10. **p < .05. ***p < .01.
